# Robust and universal predictive models for frictional pressure drop during two-phase flow in smooth helically coiled tube heat exchangers

**DOI:** 10.1038/s41598-021-99476-6

**Published:** 2021-10-08

**Authors:** M. A. Moradkhani, Seyyed Hossein Hosseini, M. Mansouri, G. Ahmadi, Mengjie Song

**Affiliations:** 1 Department of Chemical Engineering, Ilam University, Ilam, 69315-516 Iran; 2 Department of Mechanical and Aeronautical Engineering, Clarkson University, Potsdam, NY 13699-5725 USA; 3 Department of Energy and Power Engineering, School of Mechanical Engineering, Beijing Institute of Technology, Beijing, 100081 China

**Keywords:** Mechanical engineering, Chemical engineering

## Abstract

There is a lack of well-verified models in the literature for the prediction of the frictional pressure drop (FPD) in the helically coiled tubes at different conditions/orientations. In this study, the robust and universal models for estimating two-phase FPD in smooth coiled tubes with different orientations were developed using several intelligent approaches. For this reason, a databank comprising 1267 experimental data samples was collected from 12 independent studies, which covers a broad range of fluids, tube diameters, coil diameters, coil axis inclinations, mass fluxes, saturation temperatures, and vapor qualities. The earlier models for straight and coiled tubes were examined using the collected database, which showed absolute average relative error (AARE) higher than 21%. The most relevant dimensionless groups were used as models’ inputs, and the neural network approach of multilayer perceptron and radial basis functions (RBF) were developed based on the homogenous equilibrium method. Although both intelligent models exhibited excellent accuracy, the RBF model predicted the best results with AARE 4.73% for the testing process. In addition, an explicit FPD model was developed by the genetic programming (GP), which showed the AARE of 14.97% for all data points. Capabilities of the proposed models under different conditions were described and, the sensitivity analyses were performed.

## Introduction

The coiled tubes heat exchangers and steam generators have many applications in different industries, including air conditioning^1,2^, chemical processes^3–5^, refrigeration^6^, nuclear^7,8^, and others. The main reason for using this type of heat exchanger is to improve the heat transfer coefficient due to the effects of centrifugal forces without additional energy sources. In fact, the coiled tubes’ curvature creates a centrifugal force that leads to the formation of secondary flows and an increase in the heat transfer coefficient^9–11^. However, the secondary flow can lead to higher droplets’ re-deposition and waves, which leads to an increase in the pressure drop^12^. Therefore, a higher pumping power is required for these types of tubes than for straight tubes. Experimental studies show that the FPD covers about 89–95% of the total pressure drop^13^. There, the study of the frictional pressure drop during two-phase flow in helically coiled tubes is of practical interest.

### Previous studies about FPD in helically coiled tubes

#### Experimental works

There are a number of experimental works concerning the FPD in smooth coiled tubes^14–21^. Mosaad et al.^22^ studied the R134a pressure drop inside a coiled tube with vertical direction flows. They found that the refrigerant mass velocity has a significant influence on the two-phase pressure drop. Also, the higher mass flux of refrigerants leads to a higher FPD in coiled tubes. Xin et al.^23^ investigated the two-phase pressure drop of the air–water in helical tubes. Their results show that the mass flux influence on the two-phase pressure drop decreases when the tube diameter decreases. This trend was in line with that observed by Hardik et al.^18^ for water boiling.

In another work, Mozafari et al.^12^ studied the FPD of R600a in straight and coiled tubes heat exchangers. It was found that the coiled tubes have considerably larger FPD than the straight ones due to the effects of centrifugal forces. Recently, Solanki and Kumar^24^ compared the FPD of a smooth coiled tube to that of a straight tube. They reported that the FPD for the coiled tube is about 1.3 to 1.8 times higher than that of a straight tube. Wongwises and Polsongkram^25^ investigated the two-phase FPD of R134a in a vertical coiled tube with an inner diameter of 9.52 mm. They observed that the two-phase FPD in the coiled tube decreases with increasing the saturation temperature. In addition, their results showed significant effects of vapor quality and refrigerant mass flux on FPD.

Several experimental works have evaluated the two-phase FPD in steam generators^26,27^. Zhao et al.^28^ investigated the two-phase FPD multiplier in a helical tube steam generator. Their results showed that the two-phase multiplier is a function of pressure, vapor quality, and mass flow rate. Based on Santini et al.^29^ study, the FPD increases with increasing the mass flow rate and decreasing the pressure. In addition, they observed that the FPD increases with increasing the vapor quality until $x = 0.8$. Xiao et al.^30^ et al. studied the effect of curvature ratio, pressure, heat flux, and mass flow rate on two-phase FPD multiplier. It was found that the two-phase multiplier decreases with increasing the system pressure, and it is insensitive to heat flux, mass flux, and curvature ratio.

#### Previous models

The total two-phase pressure drop in coiled tubes is the sum of frictional, gravitational, and accelerational pressure drops,

1 $$\left( {\frac{dP}{{dz}}} \right)_{tp,T} = \left( {\frac{dP}{{dz}}} \right)_{tp,F} + \left( {\frac{dP}{{dz}}} \right)_{tp,G} + \left( {\frac{dP}{{dz}}} \right)_{tp,A}$$

The gravitational and accelerational terms can be calculated as following equations,

2 $$\left( {\frac{dP}{{dz}}} \right)_{tp,G} = \left[ {\alpha \rho_{v} + \left( {1 - \alpha } \right)\rho_{l} } \right]g \,{\mathrm{sin}}\left( \gamma \right)$$

3 $$\left( {\frac{dP}{{dz}}} \right)_{tp,A} = G^{2} \frac{d}{dz}\left[ {\frac{{x^{2} }}{{\alpha \rho_{v} }} + \frac{{\left( {1 - x} \right)^{2} }}{{\left( {1 - \alpha } \right)\rho_{l} }}} \right]$$

where $\alpha$ is the void fraction calculated from Abdul-Razzak et al.^31^ equation,

4 $$\alpha = \left( {1 + 0.49X_{tt}^{0.3036} } \right)^{ - 1}$$

Here, $X_{tt}$ is the Lockhart and Martinelli parameter^32^, defined as:

5 $$X_{tt} = \left( {\frac{1 - x}{x}} \right)^{0.9} \left( {\frac{{\rho_{v} }}{{\rho_{l} }}} \right)^{0.5} \left( {\frac{{\mu_{l} }}{{\mu_{v} }}} \right)^{0.1}$$

It should be noted that, for adiabatic flow inside horizontal tubes, the gravitational and accelerational terms can be ignored^33^ and $\left( {\frac{dP}{{dz}}} \right)_{tp,T} = \left( {\frac{dP}{{dz}}} \right)_{tp,F}$.

Although there are several general models for estimating the FPD in straight tubes^34–46^, the empirical models for coiled tubes are limited. As discussed previously, the centrifugal forces in helically coiled tubes can affect the FPD. Therefore, the straight tubes’ models are not reliable for estimating the FPD in coiled tubes. On the other hand, in some previous studies, the experimental data were used to propose a correlation for estimating the FPD. However, these models can be applicable only for a limited operating condition.

Most of the earlier correlations were based on the Lockhart and Martinelli^32^ separated model and modifying the two-phase multiplier,

6 $$\left( {\frac{dP}{{dz}}} \right)_{tp,F} = \phi_{l}^{2} \left( {\frac{dP}{{dz}}} \right)_{l}$$

Here the liquid phase pressure drop, $\left( {\frac{dP}{{dz}}} \right)_{l} ,$ can be calculated as

7 $$\left( {\frac{dP}{{dz}}} \right)_{l} = \frac{{2f_{l} G^{2} \left( {1 - x} \right)^{2} }}{{\rho_{l} D_{t} }}$$

where, $f_{l}$ is the liquid friction factor. For coiled tubes $f_{l}$ can be calculated by the correlation suggested by Ito^47^,

8 $$f_{l} = \left( {0.00725 + 0.076\left[ {Re_{l} \left( {\frac{{D_{c} }}{{D_{t} }}} \right)^{ - 2} } \right]^{ - 0.25} } \right)\left( {\frac{{D_{c} }}{{D_{t} }}} \right)^{ - 0.5}$$

Wongwises and Polsongkram^48^ developed an empirical model for estimating the two-phase multiplier based on experimental data for R134a condensation in vertical coiled tubes. The correlation used only the Lockhart and Martinelli parameter, $X_{tt}$, as the adjustment parameter,

9 $$\phi_{l}^{2} = 1 + \frac{5.569}{{X_{tt}^{1.494} }} + \frac{1}{{X_{tt}^{2} }}$$

They showed that their equation estimates most of the measured data with less than 20% error. However, this correlation show relatively large deviations from the Solanki and Kumar^24^ experimental data for R600a due to difference in working fluids and other operating conditions. In another work, Gupta et al.^49^ presented a modification on the Wongwises and Polsongkram^48^ correlation based on R134a experimental data using the Lockhart and Martinelli parameter and reduced pressure,

10 $$\phi_{l}^{2} = 2.76\left( {1 + \frac{7.094}{{X_{tt}^{1.378} }} + \frac{1}{{X_{tt}^{2} }}} \right)P_{red}^{0.7}$$

The same method was used by Zakeralhoseini et al.^50^ and Solanki and Kumar^24^ for fitting the R1234yf and R600a experimental data, respectively.

Several empirical correlations have been obtained based on data for coiled tube steam generators^28,30,51^. Santini et al.^29^ proposed a model based on their experimental data for a steam generator with a 12.53 mm ID and coil diameter of 0.5 m. Based on their empirical model, the FPD was a function of mass flux, specific volume, tube diameter, and vapor quality. Ferraris and Marcel^52^ developed a correlation for two-phase friction factors using experimental data from 4 sources for water-steam in vertical coiled tubes. The model was based on the homogeneous equilibrium model (HEM),

11 $$\left( {\frac{dP}{{dz}}} \right)_{tp,F} = \frac{{f_{tp} }}{2}\frac{{G^{2} }}{{\rho_{tp} D_{t} }}$$

where two-phase density, $\rho_{tp}$, can be calculated as,

12 $$\rho_{tp} = \left[ {\frac{{\left( {1 - x} \right)}}{{\rho_{l} }} + \frac{x}{{\rho_{v} }}} \right]^{ - 1}$$

The HEM method considers the same velocities for phases and assumes the flow in the tube as a pseudo single-phase flow. It should be noted that the Ferraris and Marcel^52^ model for two-phase friction factor in Eq. (11) provided a good accuracy for analyzed data and predicted 95% of data with an error of less than 21.9%. The HEM method is also used by Cioncolini et al.^51^ to estimate the FPD in the steam generators. Their database contained 679 data points for coiled tubes and 321 data for straight tubes, and the developed model showed good agreements with measured values. However, this model showed large deviations from the experimental data for high vapor qualities according to Ferraris and Marcel^52^ analysis.

As discussed above, most of the available models are developed based on experimental data for limited operating conditions, working fluids, and tube orientations. So, there is no validated model for estimating the two-phase FPD in coiled tubes for a wide range of conditions and fluids. In most experimental studies, the models for straight tubes have been used to validate the measured data. However, the centrifugal force leads to differences between FPD in coiled and straight tubes. However, intelligent methods have not yet been used to estimate FPD in coiled tubes. Therefore, this work’s primary goal is to develop a verified model for estimating the two-phase FPD in different conditions and orientations of the coiled tubes. Thus, 1267 experimental data samples from 12 different sources were gathered, covering different working fluids and operating conditions. The accuracy of the most famous correlations for estimating the two-phase FPD in straight and coiled tubes was examined using the collected experimental data. In addition, the intelligent approaches of MLP, RBF, and GP were used for developing reliable and universal models for estimating the two-phase FPD. Furthermore, the capability of the new models, as well as the earlier ones, are investigated for different operating and geometrical conditions. Finally, a sensitivity analysis was performed to understand the most influential factors on FPD in helical coils.

## Materials and methods

### Intelligent approaches

#### MLP

**Figure 1:**
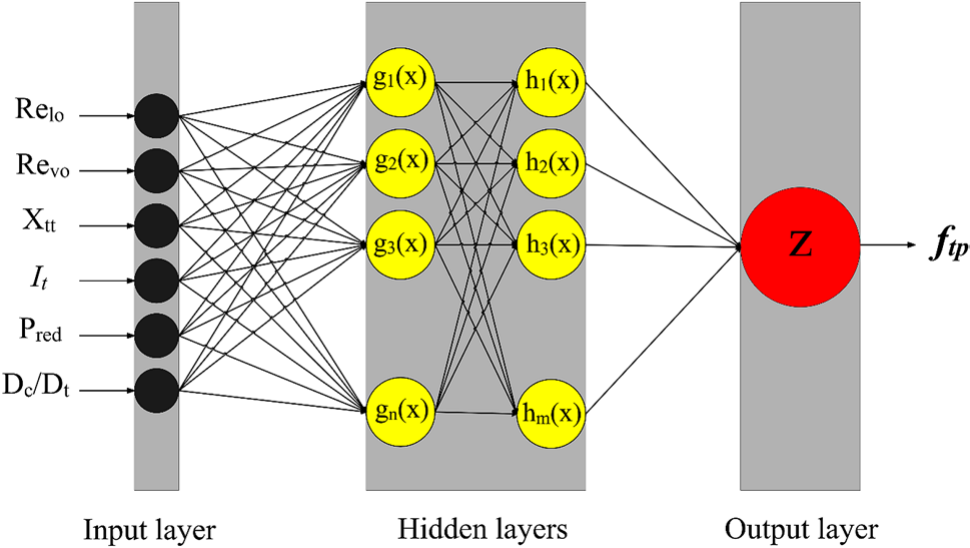
MLP network for used estimation of two-phase FPD in coiled tubes.

Recently, intelligent approaches have been widely utilized for modeling thermal and hydrodynamic characterizations of various systems^53,54^. The multilayer perceptron (MLP) is the most famous approach among several available neural networks, which has been broadly used for modeling complex systems^55–58^. Figure 1 shows the configuration of the present MLP network for modeling of two-phase FPD in coiled tubes. As observed, the network includes an input layer for taking the data, an output layer for providing an estimation associated with the input data, and ultimately one or more hidden layers as the central part of the network. Each layer in the MLP network has several neurons. The number of neurons in the input and output layers is equal to input and target parameters, respectively.

In contrast, the number of hidden layers and the number of nodes in these layers can be specified by trial and error. It should be noted that the hidden layers using the activation function send the information to the last layer of the network. The output layer displays the results received from the final hidden layer. The MLP is a useful approach for modeling complex processes that are not easy to model mathematically. The configuration details of the present MLP network are presented in Table 1.

**Table 1:** Configuration details of MLP model.

Parameters	Type/value
Number of neurons in the input layer	6
Number of neurons in the output layer	1
Number of hidden layers	2
Number of neurons in each hidden layer	15
Learning role	Levenberg–Marquardt (LM)
Train function	Trainbr
Transfer function	Tansig

#### RBF

RBF is a well-known feed-forward neural network with a fast learning process, simple structure, and high capability for modeling^59^. They use a structure similar to that for an MLP network with a single hidden layer. The total number of data samples determines the number of neurons in an RBF network. In addition, the activation functions in this network are radial basis functions. The most famous activation function used in RBF networks is Gaussian, which is defined as follow,

13 $$\psi \left( r \right) = \frac{{r^{2} }}{{e^{{2\sigma^{2} }} }}$$

where *r* is the Euclidean distance between input data and center of RBF network, and $\sigma$ denotes the standard deviation of the corresponding Gaussian function. Ultimately, the output layer presents the model outcomes as,

14 $$z = \mathop \sum \limits_{i = 1}^{n} \omega_{i} \psi_{i} \left( r \right)$$

where $\omega_{i}$ is defined as the weight of *i*th hidden neurons.

#### GP

**Figure 2:**
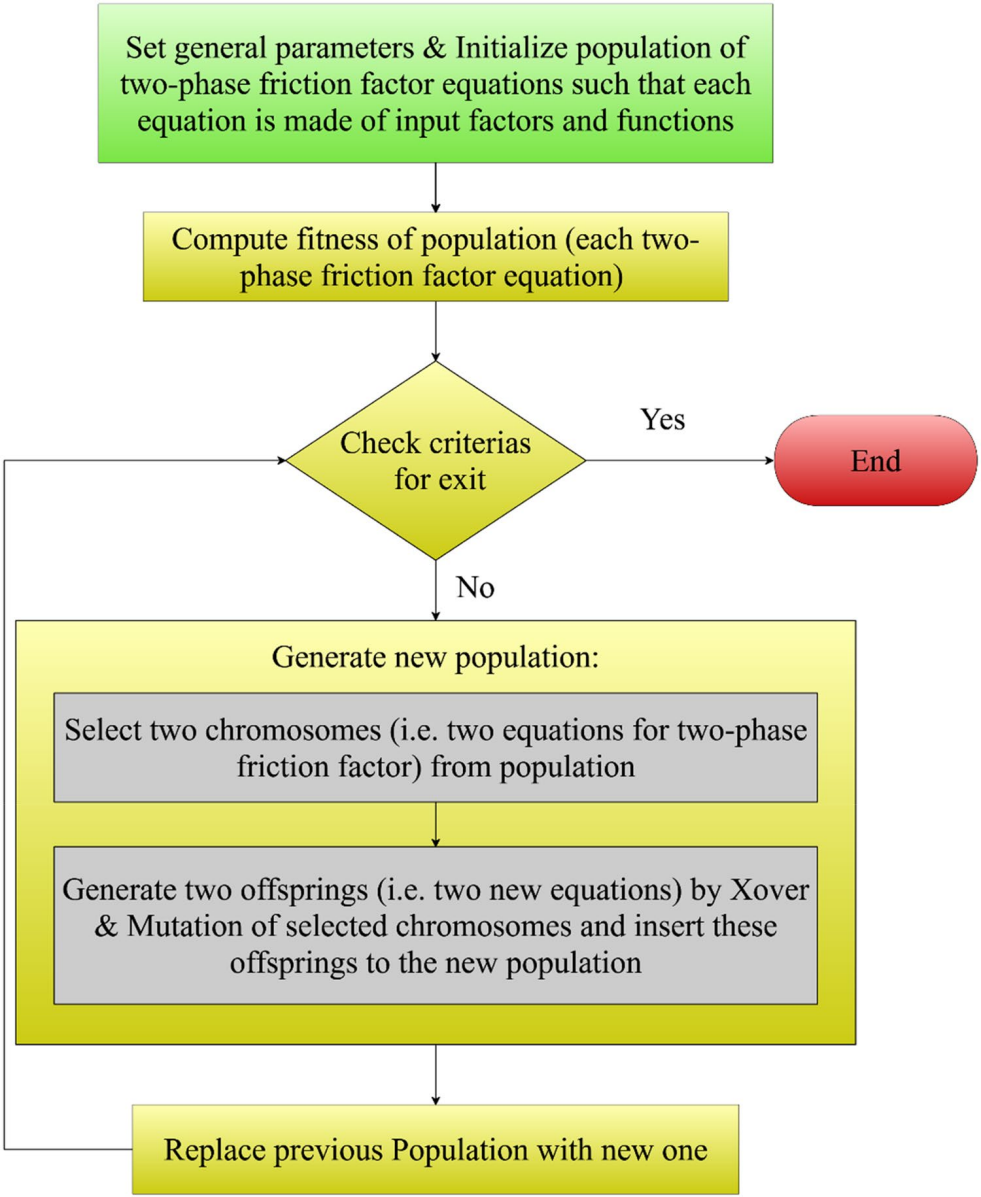
Flowchart of GP approach used for modeling of the two-phase FPD in coiled tubes.

Genetic programming (GP) is an evolutionary approach that imitates Darwin’s theory for data-driven modeling. Unlike most intelligent techniques, GP presents several explicit correlations between input variables and target function. The GP flowchart for estimating two-phase FPD in coiled tubes is presented in Fig. 2. As can be seen, the GP modeling includes four steps. In the first step, GP creates a random initial population, which each individual (chromosome) in this population is a mathematical correlation for describing the target function. In the second step, each individual's fitness is evaluated by an error metric, such as AARE. If one of the individuals satisfies the required error range, the GP process is stopped. Otherwise, two individuals, which have the best agreements with data, are selected for producing two offspring by the combination operator (Xover). These offspring change by mutation operator that leads to a change of operations or operands. This step continues until the number of individuals in the new population reaches the previous one. Finally, the previous population is replaced by the new one, and the process is repeated until the generated correlation satisfies the error metric.

In our previous works, the GP technique was used for estimating the two-phase FPD in straight channels^41^, heat transfer coefficient in straight tubes^60^, and helically coiled tubes^10^. Additional detailed information about the GP technique was reported in the earlier studies^61,62^.

### Experimental data points

There are a number of experimental studies on two-phase FPD in coiled tubes in the literature. However, some of the published papers did not provide sufficient information on the operating parameters needed for analyzing the experimental data^17,18,63,64^. In the present study, we collected and analyzed as much data as possible. As a result, a database containing 1267 experimental data points from 12 published papers covering different operating conditions for coiled tubes was collected. The operating conditions for all analyzed sources are reported in Table 2. All thermal and physical properties of the analyzed fluids are calculated with REFPROP V.9.0^65^ at the saturation conditions.

**Table 2:** The analyzed data sources for FPD in coiled tubes.

References	Fluid	Orientation	*p* (mm)	γ(Rad)	*D*_*t*_ (mm)	*D*_*c*_ (mm)	*G* (kg m^−2^ s^−1^)	*P*_*red*_ (–)	Number of points
Aria et al.^66^	R134a	Vertical downflow	45	− *π*/2	8.28	305	112–152	0.08	30
Gupta et al.^49^	R134a	Horizontal	22.50	0	8.33	90.48	100–350	0.22–0.25	93
Mozafari et al.^12^	R600a	Horizontal, inclined up flow and vertical up flow	35	0 to + *π*/2	8.30	305	155–265.5	0.16	104
Santini et al.^29^	Water	Vertical upflow	800	+ *π*/2	12.53	1000	200–600	0.09–0.27	404
Solanki and Kumar^67^	R134a	Horizontal	25	0	8.92	110	75–156	0.22–0.29	28
Solanki and Kumar^24^	R600a	Horizontal	25	0	8.92	110	75–191	0.13–0.17	54
Wongwises and Polsongkram^25^	R134a	Vertical upflow	35	+ *π*/2	8.30	305	400–800	0.12–0.19	65
Wongwises and Polsongkram^48^	R134a	Vertical upflow	35	+ *π*/2	8.30	305	400–800	0.25–0.32	48
Xiao et al.^30^	Water	Vertical upflow	NR*	+ *π*/2	12.5–14.5	180–380	400–1000	0.09–0.34	94
Yu et al.^68^	R290	Inclined downflow	NR*	− *π*/18	10	2000	224–394	0.13	20
Zakeralhoseini et al.^50^	R1234yf	Horizontal	16.70	0	8.20	95.3	95–285	0.17–0.23	60
Zhao et al.^28^	Water	Horizontal	30	0	9	292	400–900	0.03–0.14	267
**Total**				− *π*/2 to + *π*/2	8.20–14.5	90.48–2000	75–1000	0.03–0.34	1267

*Not reported.

**Figure 3:**
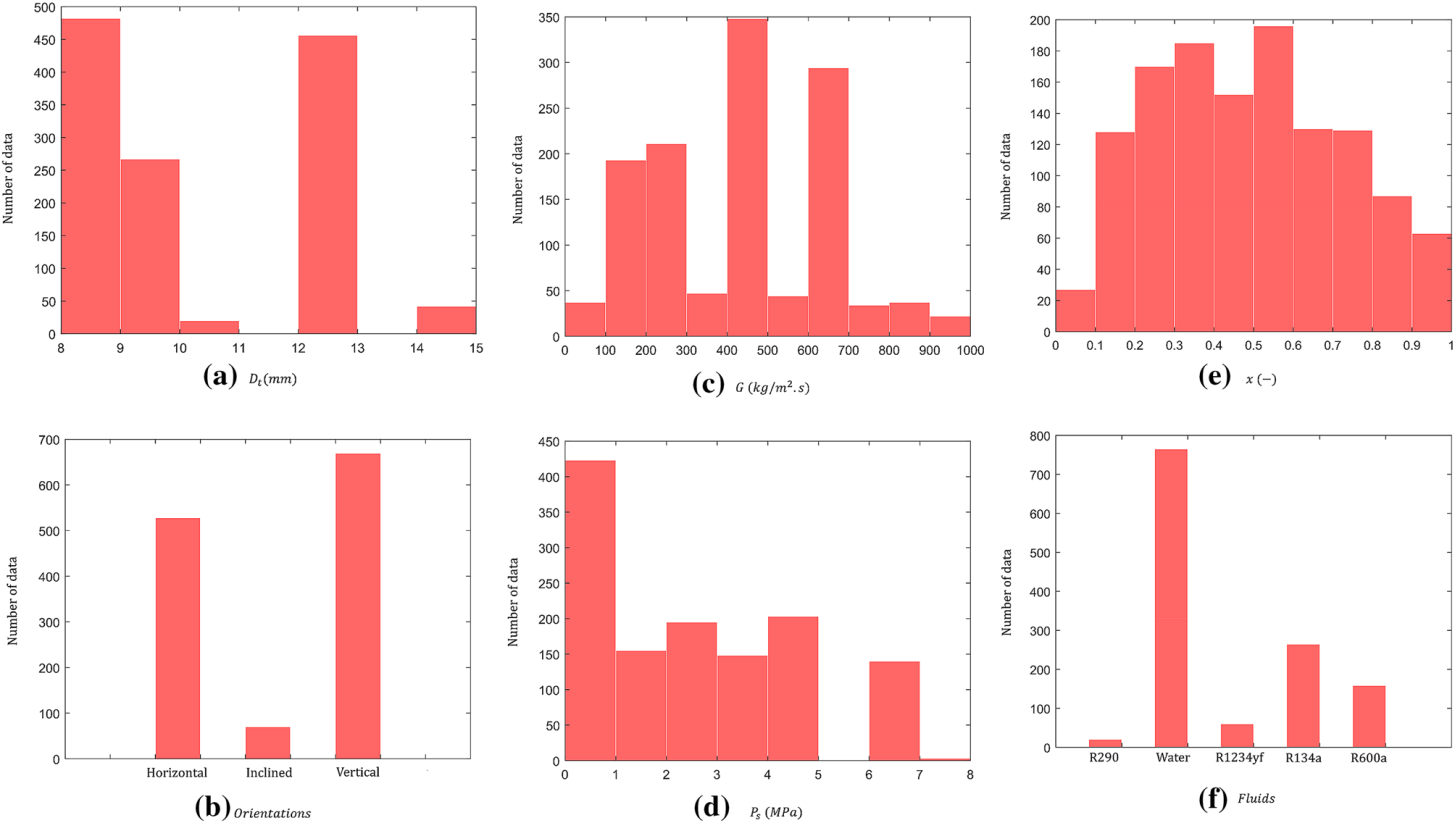
Data distribution of gathered data for two-phase FPD in helical coiled tubes.

Figure 3 shows the distribution of experimental data analyzed in this study at different operating conditions. As can be seen, the gathered data cover a wide range of operating parameters such as tube diameter, orientations, mass fluxes, saturation pressures, vapor qualities, and working fluids.

### Error analysis

In the present study, the average absolute relative error (AARE), coefficient of determination ($R^{2}$) and relative root mean squared error (RRMSE) are used to assess the models' accuracy. In addition, the arithmetic average error (AAE) is utilized to evaluate the underestimation or overestimation of different empirical models for predicting two-phase FPD. These statistical parameters are defined as,

15 $$AARE = \frac{1}{n}\mathop \sum \limits_{i = 1}^{n} \left| {\frac{{\left( {\frac{dP}{{dz}}} \right)_{F, Calc.} - \left( {\frac{dP}{{dz}}} \right)_{F, exp} }}{{\left( {\frac{dP}{{dz}}} \right)_{F, exp} }}} \right| \times 100$$

16 $$AAE = \frac{1}{n}\mathop \sum \limits_{i = 1}^{n} \left( {\frac{{\left( {\frac{dP}{{dz}}} \right)_{F, Calc.} - \left( {\frac{dP}{{dz}}} \right)_{F, exp} }}{{\left( {\frac{dP}{{dz}}} \right)_{F, exp} }}} \right) \times 100$$

17 $$R^{2} \left( \% \right) = \left( {1 - \frac{{\sum \left( {\left( {\frac{dP}{{dz}}} \right)_{F, exp} - \left( {\frac{dP}{{dz}}} \right)_{F, calc.} } \right)^{2} }}{{\sum \left( {\left( {\frac{dP}{{dz}}} \right)_{F, exp} - \overline{{\left( {\frac{dP}{{dz}}} \right)_{F, exp} }} } \right)^{2} }}} \right) \times 100$$

18 $$RRMSE \left( \% \right) = \frac{{\sqrt {\frac{1}{N}\sum \left( {\left( {\frac{dP}{{dz}}} \right)_{F, exp} - \left( {\frac{dP}{{dz}}} \right)_{F, calc.} } \right)^{2} } }}{{\frac{1}{N}\sum \left( {\frac{dP}{{dz}}} \right)_{F, exp} }} \times 100$$

## Results and discussion

### Evaluation of earlier models

As mentioned before, in some of the previous studies, a few empirical correlations were proposed. However, these correlations are not general models due to their limitations to the original set of data and operating conditions. In addition, some authors have used the straight tubes correlation for validating their experimental data. But the effect of the centrifugal forces, which are critical in coiled tubes, have been ignored. Therefore, FPD in the coiled tubes has differences from that in straight tubes. Several empirical correlations for coiled tubes and some verified general models that were developed for straight tubes are listed in Table 3.

**Table 3:** Previous models for estimating the FPD in coiled and straight tubes.

Author(s)	Correlation	Equation number	Remarks
Wongwises and Polsongkram^48^	$\phi_{l}^{2} = 1 + \frac{5.569}{{X_{tt}^{1.494} }} + \frac{1}{{X_{tt}^{2} }}$	(T3-1)	R134a two-phase flow in a coiled tube
Gupta et al.^49^	$\phi_{l}^{2} = 2.76P_{red}^{0.70} \left( {1 + \frac{7.094}{{X_{tt}^{1.378} }} + \frac{1}{{X_{tt}^{2} }}} \right)$	(T3-2)	R134a two-phase flow in a coiled tube
Zakeralhoseini et al.^50^	$\phi_{l}^{2} = 4.15P_{red}^{0.44} \left( {1 + \frac{3.119}{{X_{tt}^{1.287} }} + \frac{1}{{X_{tt}^{2} }}} \right)$	(T3-3)	R1234yf two-phase flow in a coiled tube
Solanki and Kumar^24^	$\phi_{l}^{2} = 3.17P_{red}^{0.118} \left( {1 + \frac{1.97}{{X_{tt}^{1.49} }} + \frac{1}{{X_{tt}^{2} }}} \right)$	(T3-4)	R600a two-phase flow in a coiled tube
Ferraris and Marcel^52^	$$\begin{aligned} \left( {\frac{dP}{{dz}}} \right)_{F} & = \frac{{f_{tp} G^{2} }}{{2\rho_{tp} D_{t} }} \\ \rho_{tp} & = \left[ {\frac{{\left( {1 - x} \right)}}{{\rho_{l} }} + \frac{x}{{\rho_{v} }}} \right]^{ - 1} \\ f_{tp} & = \psi f_{h} \\ \psi & = 1 + 0.207Re_{lo}^{0.27} x^{1.3} \left( {1 - x} \right)^{\frac{2}{3}} \\ f_{h} & = xf_{vo} + \left( {1 - x} \right)f_{lo} \\ f_{lo} & = 0.029\left( {\frac{{D_{t} }}{{D_{c} }}} \right)^{0.50} + \frac{0.304}{{Re_{lo}^{0.25} }} \\ f_{vo} & = 0.029\left( {\frac{{D_{t} }}{{D_{c} }}} \right)^{0.50} + \frac{0.304}{{Re_{vo}^{0.25} }} \\ \end{aligned}$$	(T3-5)	Two-phase flow in steam generators with vertical tubes
Santini et al.^29^	$$\begin{aligned} \left( {\frac{dP}{{dz}}} \right)_{F} & = K\left( x \right)\frac{{G^{1.91} }}{{\rho_{tp} d^{1.20} }} \\ K\left( x \right) & = 0.0108 - 0.00479x + 0.0387x^{2} - 0.0373x^{3} \\ \end{aligned}$$	(T3-6)	Two-phase flow in a steam generator
Xiao et al.^30^	$$\begin{aligned} \left( {\frac{dP}{{dz}}} \right)_{F} & = \phi_{lo}^{2} f\frac{{G^{2} }}{{2\rho_{l} D_{t} }} \\ f & = \frac{0.3164}{{Re_{lo}^{0.25} }}\left[ {1 + Re_{lo}^{0.053} \left( {\frac{{D_{t} }}{{D_{c} }}} \right)^{0.404} } \right] \\ \phi_{lo}^{2} & = \left( {0.377 + 6.79x - 5.66x^{2} } \right)\left( {1 + x\left[ {\frac{{\mu_{v} }}{{\mu_{l} }} - 1} \right]} \right)^{0.25} \left( {1 + x\left[ {\frac{{\rho_{l} }}{{\rho_{v} }} - 1} \right]} \right) \\ \end{aligned}$$	(T3-7)	Two-phase flow in a steam generator
Zhao et al.^28^	$\phi_{lo}^{2} = 1 + \left( {\frac{{\rho_{l} }}{{\rho_{v} }} - 1} \right)\left[ {0.303x^{1.63} \left( {1 - x} \right)^{0.885} Re_{lo}^{0.282} + x^{2} } \right]$	(T3-8)	Two-phase flow in a steam generator
Kim and Mudawar^69^*	$$\begin{aligned} & \phi_{l}^{2} = 1 + \frac{c}{X} + \frac{1}{{X^{2} }} \\ & X = \left( {\frac{dP}{{dz}}} \right)_{l} /\left( {\frac{dP}{{dz}}} \right)_{v} \\ & For\;Re_{l} \;and\;Re_{v} \ge 2000;\quad C = 0.39Re_{lo}^{0.03} Su_{go}^{0.10} \left( {\frac{{\rho_{l} }}{{\rho_{v} }}} \right)^{0.35} \\ & For\;Re_{l} \ge 2000\;and\;Re_{v} < 2000;\quad C = 8.7 \times 10^{ - 4} Re_{lo}^{0.17} Su_{go}^{0.50} \left( {\frac{{\rho_{l} }}{{\rho_{v} }}} \right)^{0.14} \\ & For\;Re_{l} < 2000\;and\;Re_{v} \ge 2000;\quad C = 0.0015Re_{lo}^{0.59} Su_{go}^{0.19} \left( {\frac{{\rho_{l} }}{{\rho_{v} }}} \right)^{0.36} \\ & For\;Re_{l} \;and\;Re_{v} < 2000;\quad C = 3.5 \times 10^{ - 5} Re_{lo}^{0.44} Su_{go}^{0.50} \left( {\frac{{\rho_{l} }}{{\rho_{v} }}} \right)^{0.48} \\ \end{aligned}$$	(T3-9)	General correlation for two-phase flow in mini and micro channels
Muller-Steinhagen and Heck^35^	$$\begin{aligned} \left( {\frac{dP}{{dz}}} \right)_{F} & = \left[ {\left( {\frac{dP}{{dz}}} \right)_{lo} + 2\left[ {\left( {\frac{dP}{{dz}}} \right)_{vo} - \left( {\frac{dP}{{dz}}} \right)_{lo} } \right]x} \right]\left( {1 - x} \right)^{\frac{1}{3}} + \left( {\frac{dp}{{dz}}} \right)_{vo} x^{3} \\ \left( {\frac{dP}{{dz}}} \right)_{lo} & = \frac{{2f_{lo} G^{2} }}{{\rho_{l} D_{t} }}\quad \left( {\frac{dP}{{dz}}} \right)_{vo} = \frac{{2f_{vo} G^{2} }}{{\rho_{v} D_{t} }} \\ \end{aligned}$$	(T3-10)	General correlation for two-phase flow inside pipes
Moradkhani et al.^41^*	$$\begin{aligned} C & = A_{0} \left( {1 - 0.193A_{1} } \right) + A_{1} A_{2} + \frac{{\left( { - 0.00183 - 0.155A_{1} } \right)}}{Bo} + A_{3} + A_{4} - A_{2} - A_{1} A_{2} A_{3} \\ A_{0} & = 1.883X + P_{red} \frac{{f_{l} }}{{f_{v} }} + 4.0834P_{red} \frac{{f_{l} }}{{f_{v} }}\sin \left( { - 0.0189Bo} \right) + \sqrt {\frac{9.491}{{XP_{red} \frac{{f_{l} }}{{f_{v} }}}}} - 1.58{\text{sin}}\left( {2.414Bo} \right) \\ A_{1} & = \sin \left( { - 19.97Bo} \right) A_{2} = \cos \left( {190.917Bo} \right) \\ A_{3} & = \cos \left( {405.278Bo} \right) A_{4} = \cos \left( {401.937Bo} \right) \\ \end{aligned}$$	(T3-11)	General correlation for two-phase flow inside mini/micro and macro channels

*Marked authors used the Chisholm^70^ method for calculating the $\phi_{l}^{2}$.

Table 4 shows the error values of the earlier models, presented in Table 3, for predicting two-phase FPD. Among the empirical correlations for coiled tubes, the ones developed by Ferraris and Marcel^52^ and Santini et al.^29^ show the best agreements with the experimental data. However, their respective AARE values of 21.96% and 25.22%, and $R^{2}$ values of 77.02 and 72.18% are far from satisfactory error range. Noteworthy that the models of Ferraris and Marcel^52^ and Santini et al.^29^ have been obtained for FPD in steam generators, which causes a high deviation for analyzed dataset. On the other hand, although the correlations proposed by Wongwises and Polsongkram^48^, Zakeralhoseini et al.^50^, Solanki and Kumar^67^, Xiao et al.^30^, Gupta et al.^49^ and Zhao et al.^28^ show a good agreement with their own respective data, they exhibit a large deviation with the measured two-phase FPD for the other data sources due to substantial differences between working fluids and operating parameters. Their total AARE values are, respectively, 31.81%, 37.75%, 37.80%, 40.02, 43.44% and 48.93%. Therefore, they cannot be considered as general and accurate correlations. The Moradkhani et al.^41^, Muller-Steinhagen and Heck^35^, and Kim and Mudawar^69^ correlations which were developed for two-phase FPD in straight tubes, show roughly similar results for helically coiled tubes with the AARE of 35.90%, 40.32%, and 42.29%, respectively. In addition, their AAE values show that the straight tubes models underestimate FPD for helically coiled tubes because these tubes have higher pressure drops than those of straight tubes due to centrifugal force and secondary flow effects^20^. Therefore, the two-phase FPD correlations obtained for the straight tubes cannot be applied for the coiled tubes. Overall, according to the results presented in Table 4, there is a critical need for developing more accurate and reliable models for estimating two-phase FPD in helically coiled tubes.

**Table 4:** Error metrics of the earlier models for predicting the two-phase FPD in coiled tubes.

Models	AARE (%)	AAE (%)	$R^{2}$(%)	RRMSE (%)	$\theta_{20\% }$(%)	$\theta_{30\% }$(%)
Muller-Steinhagen and Heck^35^	40.32	− 39.46	45.25	83.90	13.97	27.23
Kim and Mudawar^69^	42.29	− 41.22	30.52	94.50	13.65	25.10
Moradkhani et al.^41^	35.90	− 34.85	57.97	73.50	20.28	37.96
Wongwises and Polsongkram^48^	31.81	− 26.49	55.72	75.44	33.94	49.49
Gupta et al.^49^	43.44	− 33.64	16.48	103.62	20.92	35.52
Zakeralhoseini et al.^50^	37.75	− 9.62	51.95	78.59	26.76	40.41
Solanki and Kumar^24^	37.80	12.54	78.33	52.78	32.83	50.04
Ferraris and Marcel^52^	21.96	− 14.50	77.02	54.35	52.01	68.59
Santini et al.^29^	25.22	− 19.95	72.18	59.80	46.80	58.88
Xiao et al.^30^	40.02	30.65	90.17	35.55	35.83	49.09
Zhao et al.^28^	48.93	42.98	81.90	48.24	26.68	35.60

**Figure 4:**
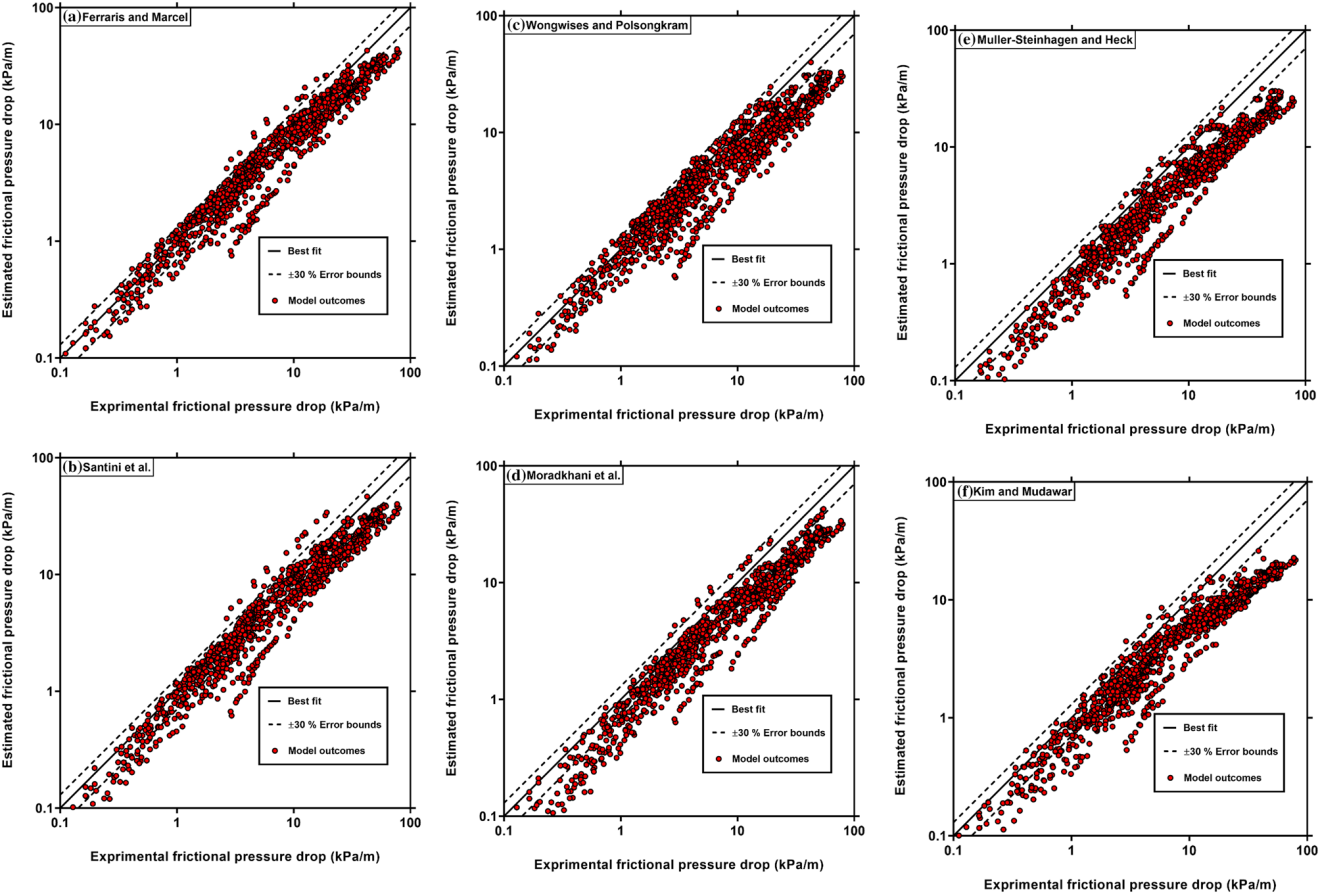
Comparison of the experimental FPD data with those estimated by the previous correlations.

For visualizing the previous models’ accuracy, Fig. 4 compares the predictions of Ferraris and Marcel^52^, Santini et al.^29^ and Wongwises and Polsongkram^48^ correlations for coiled tubes as well as the Moradkhani et al.^41^, Muller-Steinhagen and Heck^35^ and Kim and Mudawar^69^ models for straight tubes with the corresponding experimental data. These correlations were selected because they showed better results than the others models for estimating the experimental data based on Table 4. Figure 4 shows that Ferraris and Marcel^52^, Santini et al.^29^ and Wongwises and Polsongkram^48^ correlation results are more reasonable than those of straight tubes’ models, and most data points predicted by Moradkhani et al.^41^, Muller-Steinhagen and Heck^35^ and Kim and Mudawar^69^ models, are beyond the $\pm$ 30% error bounds. In addition, as discussed previously, the models for straight tubes significantly underestimate the coiled tubes’ FPD.

### Development of the new predictive methods

#### Selection of the most effective input parameters

In this study, the HEM method given by Eq. (11) is used because the secondary flow caused by centrifugal force in curved tubes boosts the two-phases’ heat and momentum transfer, which is consistent with the HEM flow hypothesis^52^. Furthermore, the HEM method uses a much simpler way to calculate the FPD compared to other methods, such as Lockhart and Martinelli^32^. On the other hand, this method's capability for modeling FPD in coiled tubes has been confirmed by several studies in the literature^29,51,52^. Accordingly, the aforementioned intelligent methods are used for developing accurate and reliable models for estimating the two-phase friction factor, $f_{tp}$ shown in Eq. (11). To achieve this goal, first the parameters that the highest effect on $f_{tp}$ should be identified.

**Figure 5:**
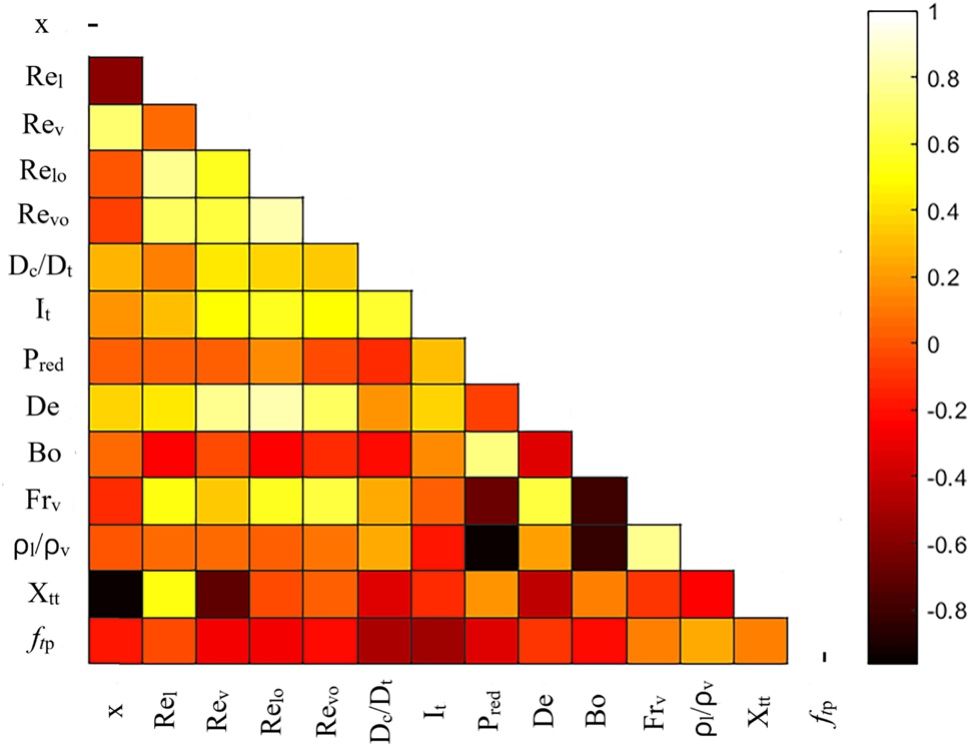
Heatmap of Spearman’s correlation coefficient between different factors.

To select the dimensionless parameters with the greatest influence on $f_{tp}$, the Spearman’s correlation coefficients^71^ between 13 probable non-dimensional parameters and two-phase friction factor are calculated, and a heatmap of the corresponding results are presented in Fig. 5. These parameters have been commonly used as models’ inputs in the previous models of two-phase FPD. As can be seen from Fig. 5, the liquid and vapor-only Reynolds numbers, i.e., $Re_{lo}$ and $Re_{vo}$ have significant correlations with $f_{tp}$, which is, consistent with Ferraris and Marcel^52^ findings. In contrast, Spearman’s method shows that the liquid-phase Reynolds number, $Re_{l}$ effect on $f_{tp}$ is insignificant. Although the vapor-phase Reynolds number, $Re_{v}$ has a high impact on the friction factor, it shows a great correlation with $Re_{lo}$ and $Re_{vo}$. Therefore, the presence of $Re_{v}$ in the model not only does not improve the accuracy of the model, but also makes the model more complex.

The coil to tube diameter ratio, $D_{c} {/}D_{t}$, tube inclination factor, $I_{t}$, reduced pressure, $P_{red}$ and Lockhart and Martinelli parameter, $X_{tt}$, present a significant correlation with $f_{tp}$, therefore, they should be used as new models’ inputs. Also using $P_{red}$ as an input parameter can take into account the effect of Phases’ density ratio, $\rho_{l} {/}\rho_{v}$ and Bond number, Bo, in the model because the reduced pressure has a high correlation with these dimensionless parameters. In addition, the influence of vapor quality, x, included in $X_{tt}$ as defined in Eq. (5). Therefore, using x and an additional model input parameter is unnecessary. In addition, the effects of both Froud number, $Fr_{v}$, and Dean number, De, on the two-phase friction factor can be satisfied by using $D_{c} {/}D_{t}$, $Re_{lo}$ and $Re_{vo}$ as input parameters.

Based on the above discussion, six dimensionless parameters are selected as the optimized input factors for modeling the two-phase FPD in coiled tubes,

19 $$f_{tp} = \frac{{2\rho_{tp} D_{t} }}{{G^{2} }}\left( {\frac{dP}{{dz}}} \right)_{tp,F} = f\left( {Re_{lo} ,Re_{vo} ,P_{red} ,I_{t} ,\frac{{D_{c} }}{{D_{t} }},X_{tt} } \right)$$

The range of dimensionless parameters used in Eq. (19) that shows the limit of applicability of the new correlation are listed in Table 5.

**Table 5:** The range of non-dimensional parameters used for modeling of FPD.

Parameter	Range of data
$Re_{lo}$, (–)	3592–143,266
$Re_{vo}$, (–)	55,143–811,688
$P_{red}$, (–)	0.034–0.325
$I_{t}$, (–)	− 1 to + 1
$\frac{{D_{c} }}{{D_{t} }}$, (–)	10.86–200
$X_{tt}$, (–)	0.006–2.76

#### New models based on neural network approaches

Based on dimensionless groups presented in Eq. (19), the neural network approaches of MLP and RBF were applied to develop accurate and reliable models to predict two-phase FPD inside the helically coiled tubes. Firstly, the models were trained using 80% of the entire data (1014 data points), and then their prediction capability was tested using the remaining data (253 points). A summary of the MLP and RBF results for testing and training processes is reported in Table 6. As can be seen, both intelligent models provide excellent results for predicting two-phase FPD in helically coiled tubes for the testing data with AAREs of 6.90% and 4.73%, and $R^{2}$ values of 99.10% and 99.63%, for MLP and RBF, respectively. Moreover, these models predict more than 87% of testing data with relative errors less than 10%. These excellent testing results confirm that the neural network-based models are accurate and reliable for forecasting the FPD in coiled tubes. Also, the input non-dimensional groups for the models are correctly selected to include the influences of different parameters on two-phase FPD. Table 6 also shows that the RBF model has slightly better accuracy in both training and testing steps, and therefore it can be selected as the most reliable model.

**Table 6:** Results of the MLP and RBF models for prediction of FPD.

Model	Process	AARE (%)	AAE (%)	$R^{2}$(%)	RRMSE (%)	$\theta_{10\% }$(%)	$\theta_{20\% }$(%)
MLP	Train	2.39	0.14	99.61	7.04	97.23	99.51
	Test	6.90	− 0.29	99.10	10.76	87.75	94.86
	Total	3.29	0.05	99.51	7.92	95.34	98.58
RBF	Train	0.08	0.00	99.99	0.41	99.80	100
	Test	4.73	− 0.26	99.63	6.84	88.14	96.44
	Total	1.01	− 0.05	99.93	3.08	97.47	99.29

**Figure 6:**
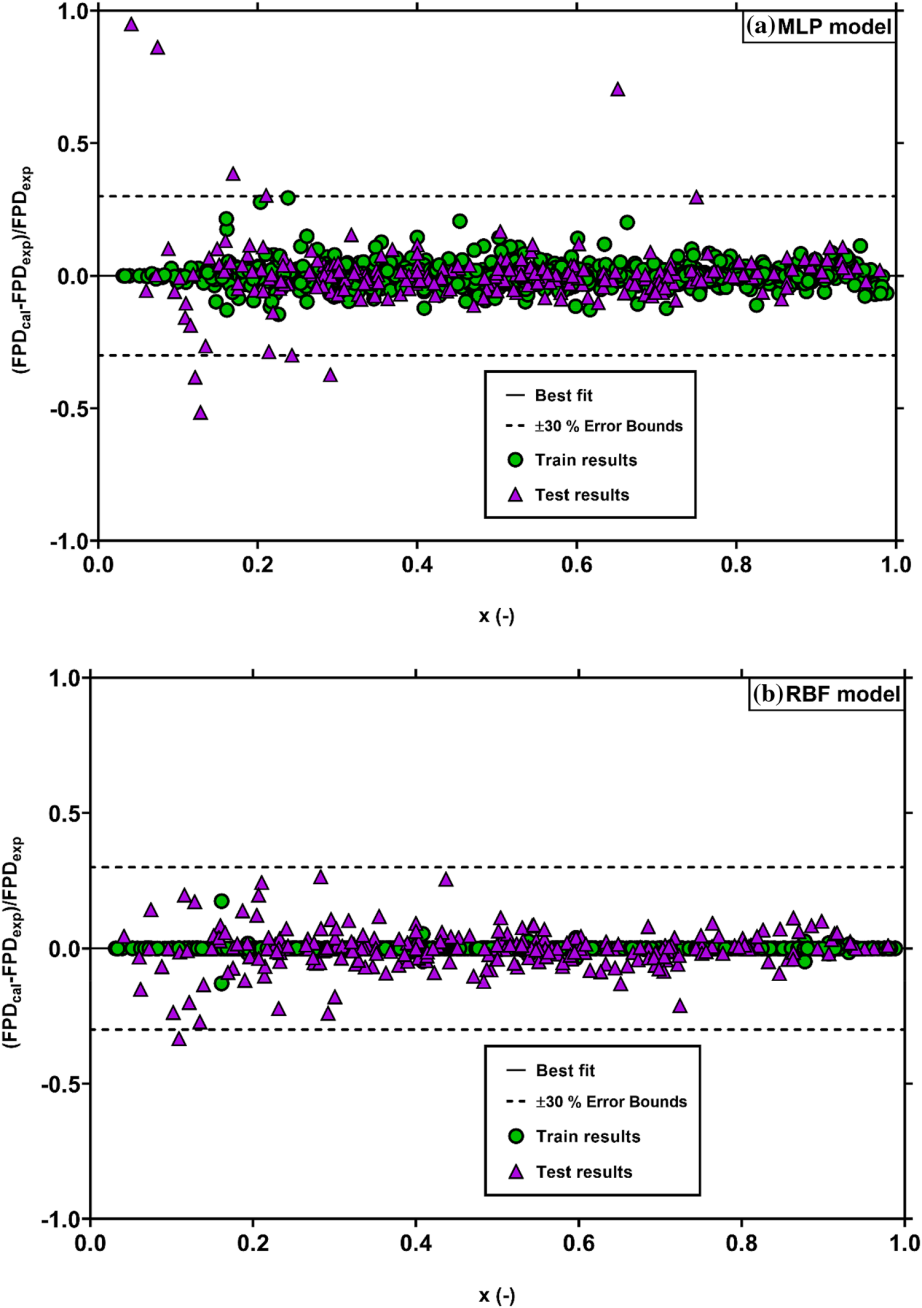
Comparison of the experimental FPD data with those estimated by the MLP (**a**) and RBF (**b**) models.

For more insight into the new models’ precision, Fig. 6 compares the outcomes of MLP and RBF models for training and test data with the corresponding experimental data. This figure reveals that although both models have good fits with actual data, the accuracy of the RBF model is slightly higher than that of the MLP model.

#### A new general correlation for FPD

In addition to the models developed by neural network approaches, the GP method was used to develop an explicit nonlinear correlation for estimating the two-phase FPD based on the HEM method. Thus, all dimensionless parameters presented in Eq. (19) were defined as GP inputs. After numerous trials using GP, the following simple, nonlinear correlation was found,

20 $$f_{tp} = 0.077 + \frac{{0.0016Re_{lo} }}{{Re_{vo} }} - 1.29 \times 10^{ - 6} \left( {\frac{{D_{c} }}{{D_{t} }}} \right)^{2} + 0.074P_{red} \left( {I_{t} - 1.67} \right) + 0.44A_{1} \left| {I_{t} } \right| - 0.043I_{t}$$

Here $A_{1}$ is given as

21 $$A_{1} = \min \left( {0.053, X_{tt} } \right)$$

**Figure 7:**
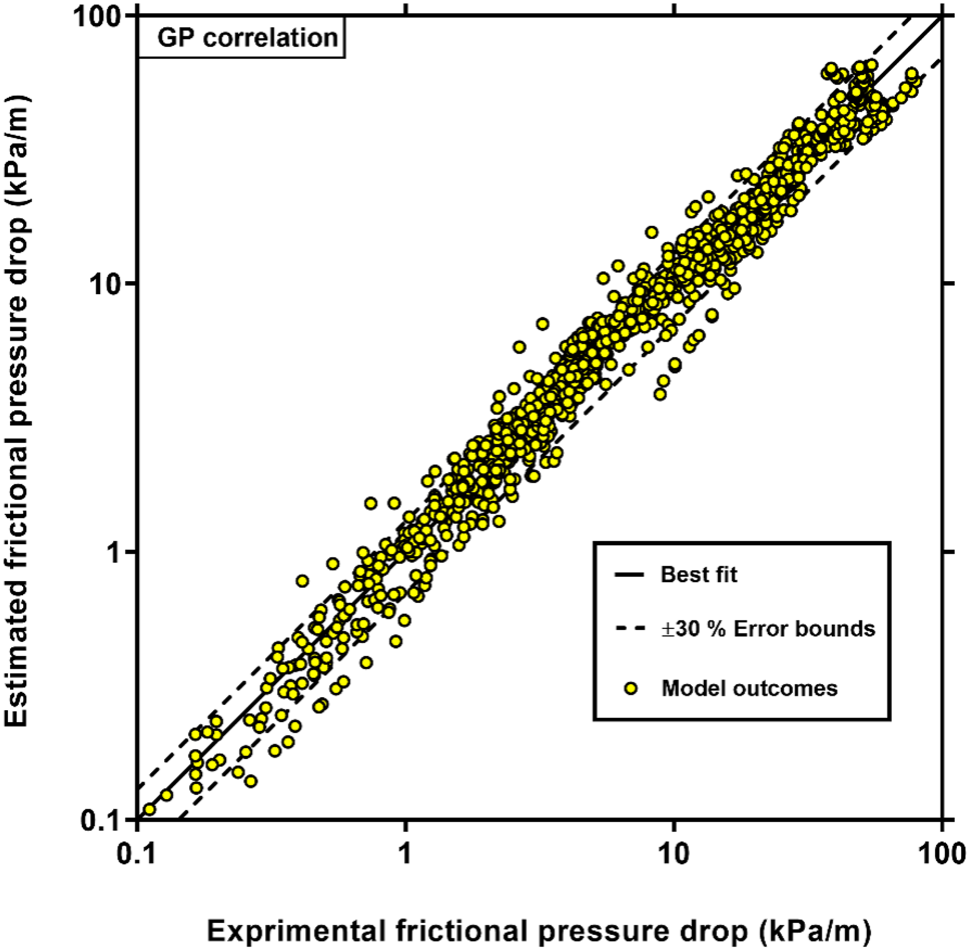
Comparison of the experimental FPD data with those estimated by GP model (Eq. (20)).

It is seen that Eq. (20) is an expression including all dimensionless parameters that are expected to affect the two-phase friction factor in coiled tubes. In order to show the accuracy of the new GP-based model given by Eq. (20) for estimating the two-phase FPD inside helically coiled tubes, the estimated values for different orientations are compared with the corresponding measured values in Fig. 7. This figure shows that the predictions of the new correlation are in good agreement with measured values. The results show that the new model estimates all data points with the AARE, AAE, $R^{2}$, RRMSE values of 14.97% and 1.83%, 92.77% and 30.49%, respectively. Furthermore, it predicts 88.08% of all analyzed data with an error of less than 30%. These good agreements with the experimental data confirm the GP-developed correlation’s ability to predict the two-phase FPD in coiled tubes at different orientations.

### Improvements of the new established models

#### Different orientations of tubes

As noted before, the earlier models were developed based on data for a specific orientation, and there is no factor for the effect of inclination angle in these correlations. However, the effect of tube orientation was considered in the new FPD models proposed in this study using inclination factor, $I_{t}$ as a dimensionless group; therefore, the new models are expected to provide accurate predictions for different flow orientations.

Table 7 presents a quantitative comparison of different correlations' accuracy for estimating the FPD in coiled tubes at different orientations. The table reveals that the best intelligent model in terms of accuracy for estimating the FPD for different orientations is the RBF model with AARE values of 1.17%, 0.64%, and 0.92% for horizontal, inclined, and vertical tubes. Also, more than 98.6% of predicted data the RBF model for all cases have an error lower than 20%. The MLP model also provides excellent results for all orientations and has AARE values of between 1.35 and 3.66% for different orientations. These perfect agreements with the measured values stem from the fact that the effect of the coil axis inclination angle is taken into account in the neural network-based models.

**Table 7:** Statistical errors of different models for estimating the two-phase FPD in coiled tubes at different orientations.

Coil axis orientation	Horizontal, 528 data pints			Vertical (upflow and downflow), 669 data points			Inclined (upflow and downflow), 70 data points		
Models	$\beta$ (%)	$\theta$ (%)	AARE (%)	$\beta$ (%)	$\theta$ (%)	AARE (%)	$\beta$ (%)	$\theta$ (%)	AARE (%)
Muller-Steinhagen and Heck^35^	3.41	13.83	46.72	21.38	37.67	35.87	22.86	28.57	34.45
Kim and Mudawar^69^	11.55	20.45	46.61	15.10	28.40	39.70	15.71	28.57	34.44
Moradkhani et al.^41^	9.09	21.97	41.30	28.10	49.18	32.42	30.00	51.43	28.50
Wongwises and Polsongkram^48^	25.95	36.55	37.85	38.86	57.70	27.90	47.14	68.57	23.64
Gupta et al.^49^	17.42	28.60	49.47	25.41	42.30	38.07	4.29	22.86	40.71
Zakeralhoseini et al.^50^	18.56	25.76	43.57	29.45	47.53	35.36	62.86	82.86	16.72
Solanki and Kumar^24^	26.89	42.99	36.88	38.12	55.01	39.20	27.14	55.71	31.31
Ferraris and Marcel^52^	30.68	49.62	27.82	69.96	84.30	16.75	41.43	61.43	27.52
Santini et al.^29^	26.33	40.91	31.53	63.53	73.84	19.27	41.43	51.43	34.58
Xiao et al.^30^	44.12	51.70	41.92	31.09	49.48	34.59	18.57	25.71	77.69
Zhao et al.^28^	48.86	63.83	32.51	9.57	13.76	58.94	22.86	31.43	77.14
**MLP model**	98.86	99.62	3.09	98.21	98.95	3.66	100	100	1.35
**RBF model**	98.67	99.81	1.17	99.70	100	0.92	100	100	0.64
**GP correlation**	67.05	85.98	17.48	76.08	89.24	13.18	71.43	92.86	13.23

Besides RBF and MLP models, the new correlation developed by the intelligent approach of GP shows satisfactory results for all orientations, and its AARE values are 17.48%, 13.18%, and 13.23%, respectively for horizontal, vertical, and inclined orientations, which shows a significant improvement in the earlier empirical correlations. Moreover, this correlation estimates 86% of data with an error lower than 30% for all orientations.

Among the available coiled tubes’ models, the results of Ferraris and Marcel^52^ and Santini et al.^29^ models for vertical orientation are acceptable with AARE of 16.75% and 19.27%, respectively. However, their AARE values for horizontal and inclined tubes are much higher. It is interesting to note that these two models were developed based on data for vertical orientation that explains their accuracy for predicting the two-phase FPD in the vertical orientation. The Zakeralhoseini et al.^50^ model also provides acceptable results for inclined tubes. However, in other cases, it still shows large deviations. The other coiled tubes’ correlations do not provide good results in different orientations. In addition, the straight tubes’ models developed by Muller-Steinhagen and Heck^35^, Kim and Mudawar^69^, and Moradkhani et al.^41^ exhibit high deviations in all orientations due to the secondary flow effects caused by the centrifugal force in coiled tubes. Overall, none of the previous models provide reasonable predictions for horizontal orientation, and all of them show the AARE of higher than 25%. Therefore, only the new models obtained by intelligent approaches can estimate the FPD in all orientations of coiled tubes with acceptable accuracy.

#### Physical trends at different operating conditions

**Figure 8:**
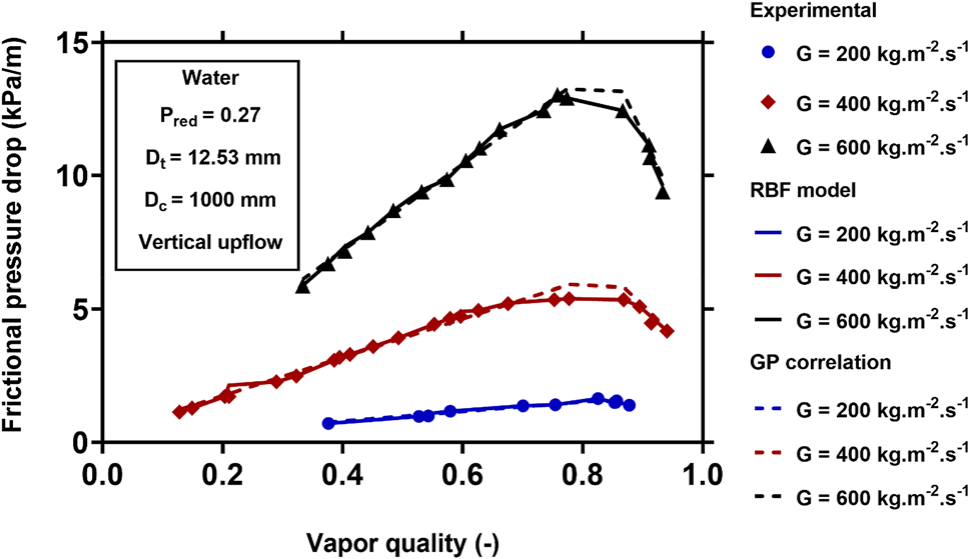
Effect of mass flux on the variation of two-phase FPD versus vapor quality. Comparisons of predictions of GP correlation (Eq. (20)) and RBF model with the corresponding experimental values^51^.

**Figure 9:**
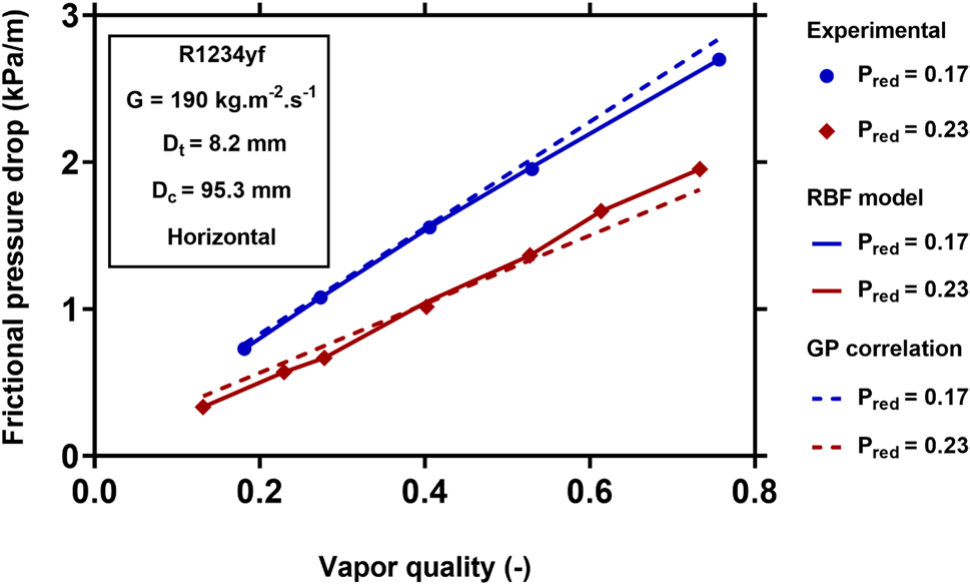
Effect of saturation temperature on the variation of two-phase FPD versus vapor quality. Comparisons of predictions of GP correlation (Eq. (20)) and RBF model with the corresponding experimental values^50^.

**Figure 10:**
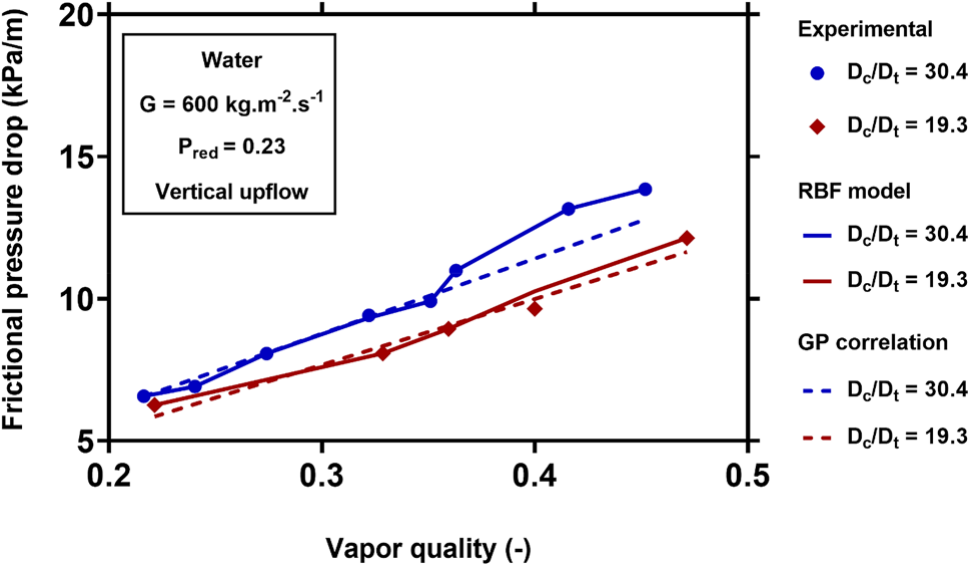
Effect coil to tube diameter ratio on the variation of two-phase FPD versus vapor quality. Comparisons of predictions of GP correlation (Eq. (20)) and RBF model with the corresponding experimental values^30^.

**Figure 11:**
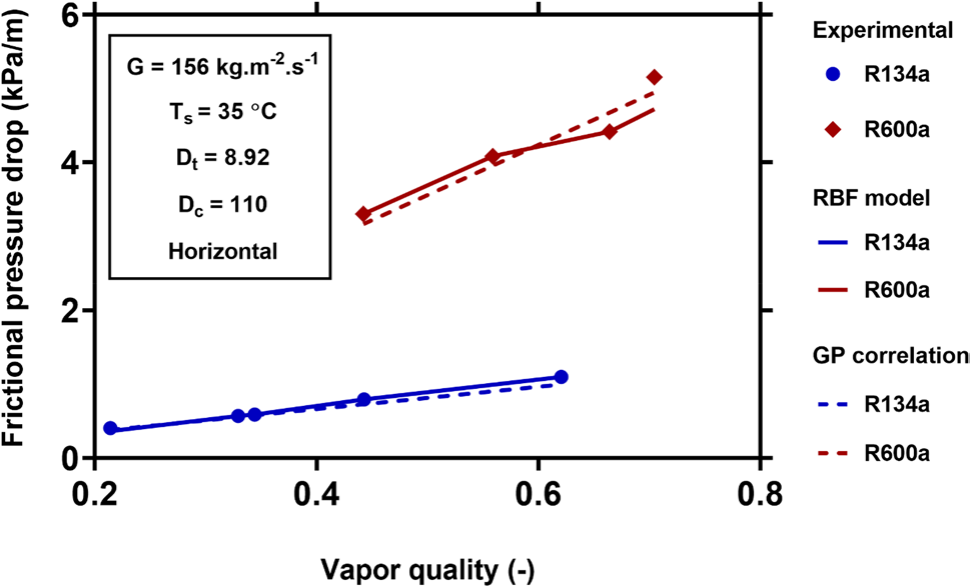
Effect of working fluids on the variation of the two-phase FPD versus vapor quality. Comparisons of predictions of GP correlation (Eq. (20)) and RBF model with the corresponding experimental values^24,67^.

For visualizing the applicability and generality of the new models for different operating conditions, Figs. 8, 9, 10 and 11 show the physical trends of the RBF model as the most reliable neural network-based model, as well as those of the new correlation established by GP. Here, the influence of vapor quality, mass flux, saturation temperature, curvature ratio, and working fluids on the two-phase FPD in coiled tubes are studied.

Figure 8 presents the influence of vapor quality and mass flux on the two-phase FPD of water in a vertical up-flow coiled tube with an inner diameter of 12.53 mm and a coil diameter of 1000 mm, at the reduced pressure of 0.27. The two-phase FPD increases with increasing the vapor quality and mass flux. In fact, a higher vapor velocity and lower liquid velocity are obtained by increasing the vapor quality. Therefore, the velocity difference at the two-phase interface increases that result in higher shear stress and FPD. On the other hand, the liquid and vapor phases' velocities increase with increasing the total mass flux. Therefore, the frictional force increases and leads to a higher FPD. Both RBF and GP models show the same trends for different values of mass flux and vapor quality. In addition, they are capable of predicting the actual data accurately.

Figure 9 illustrates the reduced pressure’s effect on R1234yf two-phase FPD in a horizontal coiled tube at the constant mass flux of 190 ${\text{kg}}\;{\text{m}}^{ - 2} \;{\text{s}}^{ - 1}$. This figure shows that the two-phase FPD decreases with increasing the reduced pressure. Increasing the reduced pressure leads to higher vapor density and lower liquid density. Therefore, the liquid velocity increases, and the vapor velocity decreases. Therefore, the two-phase velocity difference at the interface decreases, leading to lower shear stress and decreasing the FPD. Figure 9 also shows that the GP and RBF models’ predictions are in close agreement with the experimental data. Therefore, the effect of reduced pressure was properly taken into account in these models.

Figure 10 shows the influence of the coil to tube diameter ratio on water two-phase FPD at the reduced pressure of 0.23 and mass flux of 600 ${\text{kg}}\;{\text{m}}^{ - 2} \;{\text{s}}^{ - 1}$. The FPD of water reduces with decreasing the curvature ratio (increasing the tube diameter). Separations of the liquid and vapor phases are generally more difficult in the tubes with smaller diameters. Two-phase flows in smaller tubes amplify the interactions at the liquid–vapor interface, increasing shear stress and FPD. The new models capture these physics and exhibit excellent fitting with the experimental FPD.

Finally, the effect of working fluids (R600a and R134a) on two-phase FPD at the constant mass flux of 156 ${\text{kg}}\;{\text{m}}^{ - 2} \;{\text{s}}^{ - 1}$ and a saturation temperature of 35 °C is shown in Fig. 11. The experimental results show that R600a has a higher frictional pressure drop than R134a. Table 8 shows the physical properties of these fluids calculated by REFPROP v.9.0 software^65^ at the 35 °C saturation temperature. The R600a has significantly lower vapor and liquid densities compared to R134a. Therefore, R600a has higher liquid and vapor velocities, and the corresponding shear stress is higher than R134a. Therefore, the two-phase FPD of R600a is much larger than R134a. In addition, R600a has much higher liquid and vapor kinematic viscosities that is another reason for the higher two-phase FPD of this working fluid. Both the GP and RBF models show similar trends for these working fluids and have excellent agreement with the experimental data.

**Table 8:** Physical properties of R600a and R134a at the $T_{s} =$ 35 °C.

Fluids	$T_{s}$(°C)	$\rho_{l} \left( {{\text{kg}}\;{\text{m}}^{ - 3} } \right)$	$\rho_{v} \left( {{\text{kg}}\;{\text{m}}^{ - 3} } \right)$	$\nu_{l} \left( {{\text{cm}}^{2} \;{\text{s}}^{ - 1} } \right)$	$\nu_{v} \left( {{\text{cm}}^{2} \;{\text{s}}^{ - 1} } \right)$
R600a	35	537.83	11.988	0.002532	0.006481
R134a	35	1167.5	43.416	0.001473	0.002794

### Sensitivity analysis

Identifying the most important parameters affecting FPD can help the designers of helically coiled tube heat exchangers to optimize their energy efficiency. For this purpose, the gathered databank was utilized to performing a sensitivity analysis. Accordingly, the relevancy factor between each input factor ($x_{j}$) and two-phase FPD is evaluated as,

22 $$R\left( {x_{j} ,FPD} \right) = \frac{{\mathop \sum \nolimits_{i = 1}^{n} \left( {x_{j,i} - \overline{{x_{j,i} }} } \right)\left( {FPD_{i} - \overline{{FPD_{i} }} } \right)}}{{\sqrt {\mathop \sum \nolimits_{i = 1}^{n} \left( {x_{j,i} - \overline{{x_{j,i} }} } \right)^{2} \mathop \sum \nolimits_{i = 1}^{n} \left( {FPD_{i} - \overline{{FPD_{i} }} } \right)^{2} } }}$$

**Figure 12:**
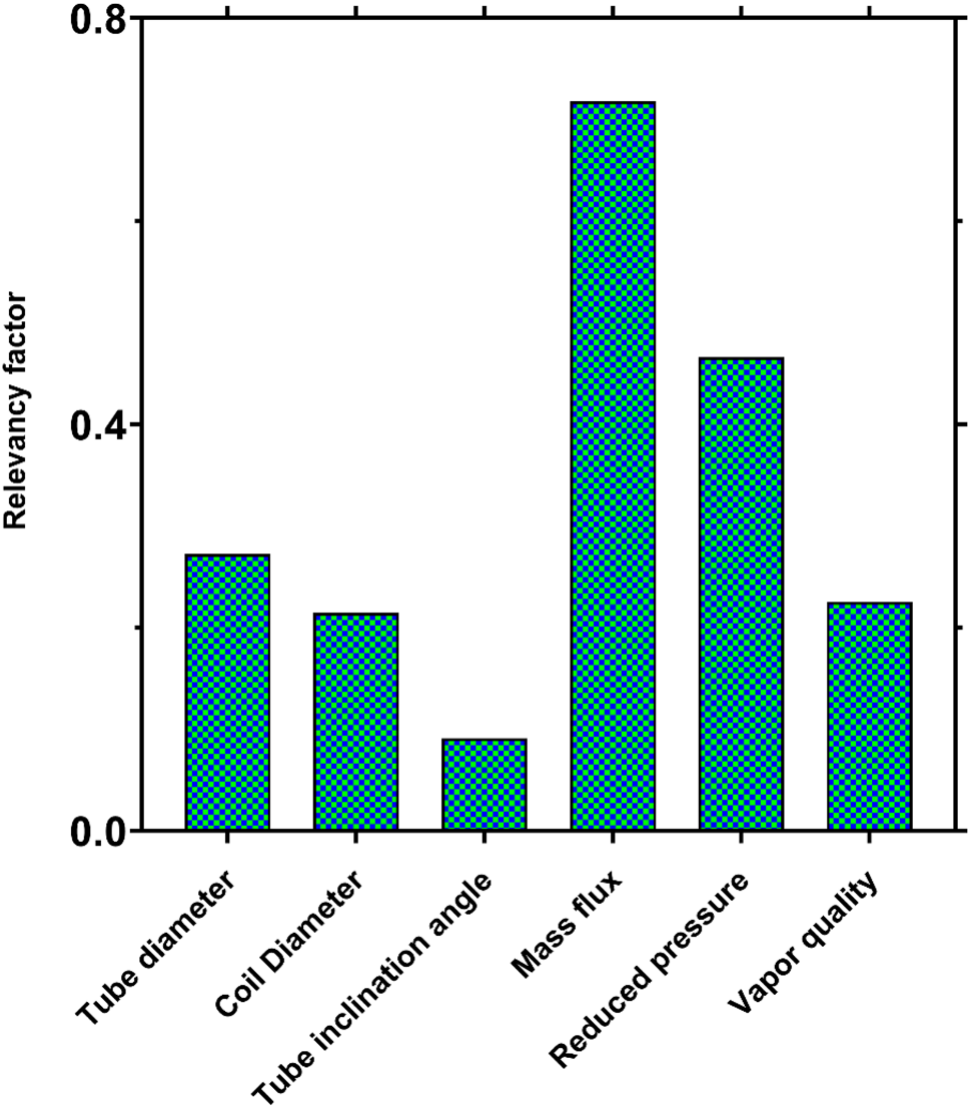
Comparing the importance of operating parameters in two-phase FPD.

Figure 12 compares the importance of different operating factors in two-phase FPD. It is seen that the flow mass flux has the most significant impact on FPD in coiled tubes, which is followed by reduced pressure, tube diameter, and vapor quality, respectively. Although the coil diameter and tube inclination angle have the lowest effects on FPD, neglecting these factors decreases the model's accuracy for predicting the FPD, which is in line with the findings depicted in Fig. 5.

## Conclusions

The intelligent approaches of MLP, RBF, and GP were utilized to develop robust and universal dimensionless correlations for estimating the two-phase FPD in helically coiled tube heat exchangers based on the homogenous equilibrium method (HEM). For validation of the models, an extensive collection of 1267 experimental data points were gathered from 12 independent studies covering different operating conditions, working fluids, and coiled tubes configurations. The main conclusions of the present study are as follows:

1. Among the earlier models for coiled tubes, the ones developed by Ferraris and Marcel^52^ and Santini et al.^29^ showed the best results with AARE values of 21.96% and 25.22%, respectively. It was found that the straight tubes correlations cannot provide reasonable predictions for two-phase FPD in helically coiled tubes due to the absence of the effects of the centrifugal forces.
2. Based on Spearman’s correlation coefficient, six dimensionless groups $\left( {Re_{lo} ,Re_{vo} ,P_{red} ,I_{t} ,\frac{{D_{c} }}{{D_{t} }},X_{tt} } \right)$ were found as the optimized input parameters for modeling two-phase FPD in helically coiled tubes.
3. Although MLP and RBF models exhibited excellent predictions, the RBF model provided the best predictions for FPD in coiled tubes for all orientations with the AARE of 1.17%, 0.64%, and 0.92% for horizontal, inclined, and vertical tubes.
4. A new universal explicit correlation was also obtained for two-phase FPD using the intelligent approach of GP, which provided much more accurate predictions than the earlier correlations with the AARE values of 17.48%, 13.18%, and 13.23%, respectively, for horizontal, vertical, and inclined orientations.
5. Among the available models, only the models proposed by Ferraris and Marcel^52^, Santini et al.^29^ for vertical tubes, and the Zakeralhoseini et al.^50^ model for the inclined tube provided acceptable results. In contrast, other previous models show large deviations from the experimental data, especially for horizontal coiled orientation.
6. The newly developed intelligent models could accurately predict the FPD in coiled tubes for different vapor qualities, mass fluxes, reduced pressures, curvature ratios, and various working fluids.
7. A sensitivity analysis based on the gathered data showed that the flow mass flux had the greatest influence on two-phase FPD in the coiled tubes. The other factors of importance in decreasing order were, respectively, reduced pressure, tube diameter, vapor quality, coil diameter, and tube inclination angle.

## List of symbols

AARE: Average absolute relative error
AAE: Arithmetic average error
Bo: Bond number, $Bo = gD_{t}^{2} \left( {\rho_{l} - \rho_{v} } \right){/}\sigma$
C: Chisholm parameter
$$D_{c}$$: Coil diameter (m)
$$D_{t}$$: Tube diameter (m)
$$f$$: Friction factor
G: Total mass flux (${\text{kg/m}}^{2} \;{\text{s}}$)
g: Acceleration due to gravity (m/s^2^)
$$I_{t}$$: Inclination factor, $$I_{t} = {\text{Tan}} \left( {\gamma {/}2} \right)$$
k: Thermal conductivity $$\left( {{\text{W/m}}\;{\text{K}}} \right)$$
La: Laplace number
p: Coil pitch, (m)
P: Pressure (Pa)
$P_{c}$: Critical pressure (Pa)
$$P_{red}$$: Reduced pressure (–)
$Re_{l}$: Liquid phase Reynolds number, $Re_{l} = G\left( {1 - x} \right)D_{t} {/}\mu_{v}$
$Re_{v}$: Vapor phase Reynolds number, $Re_{v} = GxD_{t} {/}\mu_{v}$
$$Su$$: Suratman number, $$Su = \rho \sigma D_{t} {/}\mu^{2}$$
$$T$$: Temperature (°C)
$X_{tt}$: Lockhart–Martinelli parameter, Defined by Eq. (5)
x: Vapor quality

## Greek

$\beta$: Percentage estimated within 20% deviation
$\theta$: Percentage estimated within 30% deviation
$\gamma$: Inclination angle of coil axis to horizontal, radian (0 is horizontal, $- \pi {/}2$ is vertical downflow and + $\pi {/}2$ is vertical upflow)
$$\mu$$: Dynamic viscosity $$\left( {{\text{Pa}}\;{\text{s}}} \right)$$
$$\rho$$: Density (kg/m^3^)
$\sum$: Mathematical symbol for summation
$$\sigma$$: Surface tension (N/m)
$\phi$: Two-phase multiplier
$$\upsilon$$: Kinematic viscosity $$\left( {{\text{m}}^{2} {\text{/s}}} \right)$$

## Subscripts

F: Frictional
$$l$$: Liquid
$$lo$$: Liquid only
red: Reduced
s: Saturated
$$v$$: Vapor
$$vo$$: Vapor only
